# Intraoperative Imaging Modalities and Compensation for Brain Shift in Tumor Resection Surgery

**DOI:** 10.1155/2017/6028645

**Published:** 2017-06-05

**Authors:** Siming Bayer, Andreas Maier, Martin Ostermeier, Rebecca Fahrig

**Affiliations:** ^1^Pattern Recognition Lab, Friedrich-Alexander-University Erlangen-Nuremberg (FAU), Erlangen, Germany; ^2^Siemens Healthcare GmbH, 91301 Forchheim, Germany

## Abstract

Intraoperative brain shift during neurosurgical procedures is a well-known phenomenon caused by gravity, tissue manipulation, tumor size, loss of cerebrospinal fluid (CSF), and use of medication. For the use of image-guided systems, this phenomenon greatly affects the accuracy of the guidance. During the last several decades, researchers have investigated how to overcome this problem. The purpose of this paper is to present a review of publications concerning different aspects of intraoperative brain shift especially in a tumor resection surgery such as intraoperative imaging systems, quantification, measurement, modeling, and registration techniques. Clinical experience of using intraoperative imaging modalities, details about registration, and modeling methods in connection with brain shift in tumor resection surgery are the focuses of this review. In total, 126 papers regarding this topic are analyzed in a comprehensive summary and are categorized according to fourteen criteria. The result of the categorization is presented in an interactive web tool. The consequences from the categorization and trends in the future are discussed at the end of this work.

## 1. Introduction

In neurosurgery, one of the major challenges is localization of the pathological tissue and relevant anatomical structures within the brain during surgery. The requirement for high accuracy arises due to the complex three-dimensional structure and the intraoperative deformation of the brain. Image-Guided Neurosurgical Systems (IGNS) help to overcome this challenge. Such systems register preoperative image data to an intraoperative coordinate system of the patient, in order to display the rendering of the brain structure and position of the region of interest. In the literature, numerous benefits arising from the use of IGNS are reported. These are, for example, integration/fusion of MRI/CT images and functional data, accurate localization of lesions, reduction in surgical time, the possibility of minimally invasive cranial openings, and decreased complication rates after surgery and during the stay in the intensive care unit [1–3]. To guarantee the precise localization of pathological tissue during surgery, a high rate of correlation between the preoperative image data and the patient anatomy is necessary. However, this correlation is strongly limited by the intraoperative deformation of brain tissue, the so-called brain shift phenomenon. The extent of brain shift was already described in 1986 by Kelly et al. [4]. It was observed as the displacement of small steel reference balls which were inserted along the stereotaxis surgical viewline before craniotomy. With the wide-spread use of IGNS in the operating room, brain shift gained and continues to gain more importance. Studies report that brain shift is a highly complex spatiotemporal phenomenon with a wide variety of causes, such as tissue removal during surgery, tissue swelling, loss of cerebrospinal fluid, and use of brain retractors [5–7]. The complex nature of the brain deformation appears both on the cortical surface and in the deep brain structure which does not always correlate to the direction of gravity [8]. As intraoperative brain shift is a dynamic process and shows time dependency [9], the assumption in commercial IGNS that a patient's head and brain is a rigid body [1, 8, 10] is only valid for the initial step of the surgical procedure, but not for the intraoperative situation. As a consequence, the correlation of structures identified in the preoperative image data and in the actual image data becomes incorrect, reducing the accuracy of the surgery. Thus, intraoperative brain shift may be the most significant limitation of IGNS [11].

## 2. Methods and Contribution

The purpose of this work is to review different aspects of brain shift under craniotomy, especially for tumor resection. To ensure comprehensive coverage, we first searched for publications with the term “intraoperative brain shift” in PubMed, IEEE-Xplore, and Google. The search was further restricted with the exact phrase “brain shift,” to avoid publications which only mention the words “brain” or “shift” but not in the context of “brain shift.” In order to cover all relevant aspects of this topic, we searched for publications which related to at least one of the following aspects, measurement, quantification, compensation, modeling, or registration. In this work, we only consider intraoperative brain shift under craniotomy, especially for lesion removal surgery. Thus, publications focusing on Deep Brain Stimulation (DBS), which is a minimally invasive therapeutic procedure, and papers dealing with Diffusion Tensor Imaging (DTI), which is often used for preoperative planning but does not fit the real time constraint in lesion removal surgery, are excluded.

To ensure that the term “intraoperative brain shift” is distinct and no other papers are published with its synonyms such as “intraoperative brain deformation,” “intraoperative brain distortion,” and “intraoperative cerebral surface deformation,” we also searched for papers on PubMed, IEEE-Xplore, and Google with these synonyms but without the term “intraoperative brain shift.” No additional papers were found.

In this work, we reviewed papers published before February 2016. In total, approximately 2600 publications were found. The aim is to provide a review which covers several aspects of the topic, while ensuring the popularity, topicality, and meaningfulness of the selected papers. Therefore, we do not only include all publications in Pubmed and IEEE-Xplore but also the first 100 most relevant papers in Google Scholar. Google Scholar sorts the publications via Page Rank algorithm [12], which means the directly and indirectly referenced publications have the highest rank. After we deleted the duplicated publications and papers which do not contain “brain shift” and “brain deformation” in the abstract or use these terms as keyword, 126 papers remained. This database with 126 papers forms the basis of this review.

In this review, we focus on the clinical experience of intraoperative imaging modalities and compensation methods of brain shift especially in tumor resection surgery. First, we survey the state-of-the-art intraoperative imaging systems, which are commonly used to correct brain deformation. Since the measurement and causes of brain shift for tumor resection are not the focus of this review, these aspects are only introduced briefly. Different compensation methods are presented considering mathematical aspects and compared to each other. To conclude the discussion, the trends and consequences from the categorizations of the reviewed publications are discussed. To avoid overlap with the review published by Gerard et al. [13] which is focused on the causes and measurement of brain shift, we categorized the literature from a mathematical and algorithmic point of view. The fourteen categories of interest are brain shift compensation strategy, global transformation model, local transformation model, computational platform, registration basis, optimization method, similarity metric, intraoperative modality, constitutive model type, mesh element, quantification object, validation, and treatment (the categories in [13] are physical, surgical, biological, intraoperative imaging, other, registration, and modeling. This categorization focuses on the causes of brain shift rather than on the algorithmic approaches to identify and correct for brain shift).

In total, 116 publications are grouped by the defined fourteen categories. With the support of the Technical University of Munich, the categorization of the 116 publications is presented as an interactive web tool (http://livingreview.in.tum.de/intraoperative_brain_shift/). The remaining ten papers could not be classified in the above categories: two publications describe the clinical use of IGNS [14] and its application to correct for brain shift [15]. A third publication discusses the biomechanical behavior of brain tissue [16]; a fourth proposes the total Lagrangian formulation of Finite Element method for computing organ deformation in [17]. Two papers focus on the determination of the position and orientation of an ultrasound image in the preoperative image coordinate system [18, 19]. A seventh publication aims at the reconstruction of superficial vessels of brain which can be used to compensate for the brain shift by [20]; an eighth presents a registration and interactive visualization framework based on a remote high-end computer with a maximum of graphics capacity in a two-page abstract [21]. A ninth [22] presented an image-based reregistration scheme between preoperative MR and intraoperative US to provide accurate rigid patient registration and finally a review paper with the focus on soft tissue modeling [23].

## 3. Clinical Experience with the State-of-the-Art Intraoperative Imaging Modalities

Since commercial IGNS assumes the patient's head is a rigid body and the intraoperative navigation is based on preoperative CT- or MR-datasets, intraoperative brain shift, which is a nonrigid deformation of the brain tissue, influences the accuracy of the surgery result. If the surgeon does not have real time image data of the complex anatomical structure of the brain, the benefit of IGNS may turn into an increased risk for the patient. Commercial intraoperative imaging techniques, such as intraoperative magnetic resonance imaging (iMR) and intraoperative ultrasound (iUS), provide the neurosurgeon with the essential intraoperative image data. The clinical experience with image-guided neurosurgery systems combined with intraoperative imaging devices, either iMR or iUS, has been reported [9, 24–42] and will be reviewed in the following section.

### 3.1. Intraoperative MR

It is a very challenging task to determine the magnitude and direction of brain tissue deformation during surgery. The introduction of intraoperative magnetic resonance imaging into neurosurgery in 1994 [26] opened new opportunities to increase the accuracy of neurosurgical procedures by providing frequent image updates with high soft tissue resolution for the neurosurgeon. These images can be used to estimate the intraoperative brain shift in real time.

The most common use of intraoperative MR updates image data during neurosurgery, to assess the extent of the tumor resection and to identify surrounding functional structure to minimize morbidity after the intervention [33]. One of the first experiences with iMR was reported in [38]. The authors described the surgical setting of intraoperative MR in a twin operating theater, known as the “Heidelberg concept,” combining a conventional operating room with a radio-frequency-shielded operating room, containing an open low-field 0.2 T MRI scanner with a static magnetic field. Twenty-seven patients underwent neurosurgical procedures, such as biopsy and tumor resection in this iMR operating environment. The surgical interventions were performed in the standard operating room and with intraoperative control provided at intervals using the iMR scanner. The image quality of standard sequences, such as T1 and T2, was acceptable. However, compared to a scanner with superconducting magnets, the scanning time required to achieve comparable results is longer, because the number of acquisitions has to be increased [38]. The benefits of such a system include accurate localization and targeting of the tumor with minimal resection of normal brain tissue, observation of brain shift in serial images, and information redundancy when using fiducial markers.

Other clinical studies that either describe the development and application of iMR in neurosurgical procedures [24, 26] or evaluate the clinical usage and outcomes of iMR [25, 26, 28, 29] have shown that the integration of iMRI in a neurosurgical procedure helps the surgeon to reduce the tumor residual and leads to a high rate of complete resection. A summary is given in Table 1. Hadani et al. [24] implemented a concept similar to [38] and report elimination of inaccuracies from brain shift. Black et al. [29] found that their low-field, 0.5 T iMR system, which is designed with coils in two separate but communicated cryostats, offered several advantages combined with image guidance systems. Knauth et al. [25] showed that the integration of low-field iMR in intracranial high-grade glioma operations increased the extent of the tumor resection significantly. The safety, efficacy, and functionality of low- (0.2 T) and high-field (1.5 T) iMR scanners were analyzed by [26, 27]. They demonstrated similar experiences as [29, 38] by using iMR in various neurosurgical procedures such as brain biopsy, tumor resection, and cyst drainage. In addition, the high-field system also benefits from functional techniques including MR spectroscopy, functional MRI, MR angiography, chemical shift imaging, and diffusion-weighted imaging. Both technologies allow compensation for intraoperative brain shift [26, 27] and lead to increased extent of tumor resection [9]. Nimsky et al. [28] analyzed the feasibility of the image update procedure with iMR in a group of patients undergoing craniotomy for brain tumor surgery. The tumors were microscopically completely removed in 14 out of 16 cases. Thus, iMR image data compensate for the effects of brain shift with a high degree of accuracy. Updating the neuronavigation system with intraoperative MR images seems to be the most reliable way to compensate for intraoperative brain shift [28].

### 3.2. Intraoperative US

Ultrasound has been used as an intraoperative instrument since the 1980s [32]. In contrast to intraoperative MR, the most important advantage of intraoperative US is that it provides inexpensive image data in real time [28, 31]. The mapping of preoperative MR and intraoperative US image data can be used to evaluate the effects of registration error and tissue movement on the overall accuracy of IGNS [39]. Lunn et al. validated the usability of iUS in three in vivo porcine experiments [42]. The quantitative analyses show that coregistered iUS could measure the intraoperative displacement effectively and could be used with a computational model. Due to its poor image quality, iUS has not entered common use in neurosurgery. However, with improved image quality and new technical developments such as three-dimensional ultrasound, iUS imaging has seen a revival in the last years. White et al. [41] designed, constructed, and tested an intraoperative transcranial ultrasound monitor via shear mode in ten healthy human subjects, which utilizes shear wave propagation instead of longitudinal waves through the skull. Linear regression analysis shows that the localization of brain structures with the new shear mode ultrasound monitoring system correlates well with MRI-based localization. The introduction of three-dimensional ultrasound has increased the value of neuronavigation substantially, making it possible to update intraoperative images several times during surgery and thereby minimize the problem of brain shift [30, 35]. For example, Gronningsaeter et al. [36] describe a system that integrates a high-end three-dimensional ultrasound system into a neuronavigation system, the SonoWand. This system can be used not only as a stand-alone ultrasound, but also as a conventional preoperative MRI- or CT-based neuronavigation system. Keles et al. [31] reported that the coregistration accuracy of preoperative MR and intraoperative US achieved 1.36 mm. Ohue et al. [32] evaluated brain shift effectively by comparing real time US images with corresponding preoperative images. Compared to iMR systems, iUS is more time effective [35] because the intraoperative images of patients are acquired in situ. The expenditure of time for one intraoperative US data set is only 5 minutes [30]. El Beltagy and Atteya [40] demonstrate the benefits of intraoperative ultrasound during resection of fourth ventricular tumors in children. They were able to detect and correct tissue shifts at any time during stepwise tumor resection, update new scans, and verify new tumor size by a neuronavigated pointer. In general, the fusion of intraoperative two- or three-dimensional ultrasound images with MRI makes perception of available information easier by providing both updated image data and an extended overview of the operating field during surgery [34]. It is reliable, accurate, and easy to use and provides a continuous real time feedback without interrupting surgery [88].

## 4. Measurement of Intraoperative Brain Shift 

Since intraoperative brain shift is the most significant source of error in image-guided craniotomy, the understanding of this phenomenon is a crucial step towards its modeling and compensation. Two different approaches are commonly used to measure and quantify magnitude and direction of brain shift. One is direct measurement which compares the preoperative MR image data with the data acquired directly on the brain surfaces of the patient during the surgery. A coagulation device such as the ACUSTAR may be used [1, 46, 48]. The second approach is to analyze the pre- and intraoperative image data based on registration procedures [5, 8, 43–45, 47]. If pre- and intraoperative MR data are used, gradient echo images are acquired. That is because of the high resolution and high readout gradient [8, 44] of gradient echo image. These result in relatively little geometric distortion in the readout direction caused by B0 inhomogeneity [44].

Independent of the availability of intraoperative image data, various questions concerning the magnitude, direction, and sources of brain shift have been investigated by several groups [1, 5, 8, 9, 11, 43–47].  Table 2 categorizes these ten publications according to the imaging modality, global transformation method, registration basis, similarity measure, and quantification objective. The first quantitative studies of brain shift analyzed the magnitude and direction of the deformation of the dura and brain surface between imaging and surgery [46], at two time points separated nearly by one hour after the dura opening but before the tumor resection [1] or before and after dura opening [48]. With the development and application of iMR and iUS, quantitative analysis of subcortical deformation was facilitated. Maurer et al. [43] used a 1.5 T iMR to investigate the magnitude of brain shift during and after surgery. An extension of this work was published by Hill et al. [44] which provides more quantitative results and includes the analysis of 3D deformation maps. Low-field iMR was used in [5, 9] to compare pre- and intraoperative three-dimensional images, allowing evaluation and visualization of the extent of cortical surface and subcortical structure. Hartkens et al. [8] investigated the predictability of brain shift by comparing the iMR images at the start and end of the surgery. Deformation patterns were analyzed quantitatively with respect to the magnitude and direction. In order to evaluate and analyze the maximum deformation on the cortical surface and in the subcortical structure, different strategies were applied in [45]. Letteboer et al. [47] used iUS data and compared it to the preoperative MR data in order to analyze the magnitude and direction of brain shift. This study also shows that the three-dimensional ultrasound data is feasible to measure the intraoperative brain shift. An exception to the above categorization is a retrospective study in 2005 [11]. In this study, the authors determine influences of various factors such as tumor size, periventricular location, patient age, prior surgery or radiation therapy, patient positioning, use of medication (e.g., mannitol), and length of operation time, on intraoperative brain shift. Pre- and postoperative gadolinium enhanced T1-weighted MRI data are statistically compared to validate whether the factors mentioned above correlate with the success of tumor resection using image-guided techniques.

### 4.1. Result of Quantitative Analysis of Brain Shift Phenomenon

As the measurement and causes of brain shift are not the focus of this review, only a short summary of the quantitative analysis of brain shift will be provided. The interested reader is directed to [13] for a detailed overview of causes and quantification.

Brain shift is a slow, time dependent phenomenon [5] and changes continually during the surgery [9]. A significant deformation can be observed after the dura is opened due to release of intracranial pressure alone. The magnitude of this effect is typically a few mm (e.g., up to 10 mm in [1, 46] and 13.4 mm in [47]). After the dura is opened, the displacement of the brain increases continuously but slowly [46]. Of course tumor resection and tissue manipulation increase the magnitudes of cortical and deep tumor margin deformations dramatically. Deformation after resection is highly variable and depends on the volume of tissue removed [11] with values as high as 23.8 mm reported [5]. Statistical analysis shows that brain shift does not show significant correlation with patient age, mannitol dose, fluid volume change, partial pressure of arterial carbon dioxide, prior surgery, or radiation therapy [1, 5, 11]. In contrast to cortical surface and deep tumor margin, midline shift is much smaller: in some cases of the study by [44] the midline does not shift. Even tumor resection causes only minor deformation in the midline [43].

After the dura opening, the direction of intraoperative brain shift is both sinking of the brain surface and bulging [1]. The relationship between patient position and brain shift is complex. The study by [5] claims that the direction of brain shift was influenced primarily by patient and head positioning, but it has no significant effect on the amount of brain shift. The measurement by [47] shows that the angle between the main direction of shift and gravity is on average 60°, with a maximum of 88°.

## 5. Compensation for Intraoperative Brain Deformation

As one of the most important error sources in IGNS, intraoperative brain shift must be compensated in order to increase the accuracy of neurosurgery, especially in the case of nonminimally invasive craniotomy, because the extent of brain shift depends primarily on the size of the craniotomy and the duration of the surgery [59]. Basically, there are two different strategies to compensate for intraoperative brain shift. The first is to use registration techniques based on intraoperative image data. Modalities such as iMR and iUS but also Laser Range Scanner (LRS) and Stereo Vision are used to acquire intraoperative image data [49–52, 56, 59–70, 77, 79, 80, 87]. Usually, LRS and Stereo Vision are used to acquire the intraoperative cortical surface deformation which is then integrated into a precomputed patient specific biomedical model in order to estimate the volumetric deformation. However, these papers [67–70, 77, 79, 80] are still categorized as registration-based method, because they only presented the registration approaches which register the intraoperative surface data either with preoperative MR or between data acquired at different time points. The model-based purpose is not described directly in these works; therefore the purpose of these works is not clear without any additional knowledge. Nevertheless, it should be kept in mind that once LRS or Stereo Vision are used as intraoperative modalities a model-based strategy is commonly pursued. An alternative strategy is to build a computational model (e.g., a Finite Element Model) of the brain based on constitutive constraints, which describe the stress-strain response of the tissue under various loading conditions. This model is combined with sparse intraoperative image data to update preoperative images [45, 53–55, 57, 58, 71–76, 81–86]. A summary of the compensation techniques for brain shift is shown in Table 3. Various aspects of image registration-based and model-based compensation strategies are explained in the following section.

### 5.1. Fundamentals of Nonrigid Registration of Medical Images

The fundamental challenge of compensation for intraoperative brain shift is to find the optimal geometric transformation *T* : (*x*, *y*, *z*) ↦ (*x*′, *y*′, *z*′) which maps the source image to the target image. Since the brain consists of elastic tissue, finding the optimal image to image or image to model, nonrigid registration method considering boundary conditions, such as craniotomy size and tumor size, is the most crucial task.

Nonrigid registration methods can be categorized by their transformation model, registration basis, or similarity measurement. Different techniques which are found in our literature database are introduced here briefly.

#### 5.1.1. Transformation Model

There are two ways to model a nonrigid registration. The first method is to model the transformation in a parametric fashion by using a set of unknowns and the second is to describe the deformation at every voxel in a nonparametric fashion. Typically, the number of parameters used in the parametric models is much smaller than the number of voxels in the nonparametric models.

*Parametric Transformation Models.* Parametric models based on splines such as Thin Plate Splines and Free Form Deformation with *B*-splines are commonly used. Assume a set of corresponding feature points exist in the source and target image. The location of the feature points in the target image can be mapped onto its counterparts in the source image by using splines to either interpolate or approximate the displacements. Splines provide a smooth approximation of the displacement field between the feature points. For the transformation function *T* : *ℝ*^*d*^ → *ℝ*^*d*^ with *d* as dimensionality of the images to register, the interpolation condition can be written as

(1) $$\begin{array}{c} T\left(\;p_{i}\;\right)=q_{i},\;i=1,\ldots ,n, \end{array}$$

where **p**_*i*_ ∈ *ℝ*^*d*^ and **q**_*i*_ ∈ *ℝ*^*d*^ denote the location of the feature points in the source and target image.

(i) * Radial Basis Functions (RBF)* are defined as radially symmetric functions *R*(**x**) = *R*(‖**x**‖) in which the value depends only on the Euclidean distance of the argument from the origin **c** [130]. RBFs construct a linear function space which depends on the position of the known data points to an arbitrary distance measure [131].
(ii) *Thin Plate Splines* [132] is a subfamily of Radial Basis Functions. The concept of Thin Plate Splines is based on the theory of deformation of thin elastic plates, where the bending forces are orthogonal to the surface. The superposition of the bending forces must be zero; otherwise the thin plate will shift.
The Thin Plate Spline is defined as a linear combination of the sum of *m* basis functions and a weighted sum of a set of *n* arbitrary Radial Basis Functions:
  (2) $$\begin{array}{c} t\left(\;p_{i}\;\right)=\overset{m}{\underset{k=1}{\sum }}\alpha_{k}\phi_{k}\left(\;p_{i}\;\right)+\overset{n}{\underset{j=1}{\sum }}\beta_{j}R\left(\;r\;\right), \end{array}$$
where *ϕ*_*k*_(**x**) is the *k*th basis function and **α** and **β** are vectors with coefficients which define the Thin Plate Splines. The Radial Basis Function of the Thin Plate Spline *R*(*r*) is defined as
  (3) $$\begin{array}{c} R\left(\;r\;\right)=\left\{\begin{array}{cc} r^{2}\ln r & \text{in }2D\\ r & \text{in }3D \end{array}\right. \end{array}$$
with *r* = ‖**p**_*i*_ − **p**_*j*_‖_2_ as the distance between the control points and the point under consideration. Inserting (2) into (1) and solving for the coefficients **α** = (**α**_1_^⊺^,…, **α**_*m*_^⊺^)^⊺^ and **β** = (**β**_1_^⊺^,…, **β**_*n*_^⊺^)^⊺^ yield a Thin Plate Spline transformation. To guarantee the uniqueness of the solution the following constraints formulated in (4) must be considered: since the bending forces are orthogonal to the control points and the sum of the forces is zero, the coefficients **β** must sum up to zero and their inner products with the coordinates of the control points are also zero.
  (4) ∑j=1nβj=0,∑j=1nβjϕkpi=0,k=1⋯m.
Thus, (2) can be expressed in matrix form as
  (5) $$\begin{array}{c} \left(\begin{array}{cc} R & P\\ P^{⊺} & 0 \end{array}\right)\left(\begin{array}{c} \beta \\ \alpha \end{array}\right)=\left(\begin{array}{c} Q\\ 0 \end{array}\right), \end{array}$$
where **R** ∈ *ℝ*^3*n*×3*n*^ is a symmetric distance matrix and **P** ∈ *ℝ*^3*n*×3*m*^ is a matrix given by *P*_*ik*_ = *ϕ*_*k*_(**p**_*i*_) and **Q** = (**q**_1_^⊺^,…, **q**_*n*_^⊺^)^⊺^. Once the coefficients **α** and **β** are known, each point on the source image can be transformed via (2). This equation can be interpreted as follows: in a three-dimensional case, the first term is the linear part which defines a volume that best matches all control points; the second term corresponds to the bending forces provided by *n* control points.
(iii) The basic idea of* Free Form Deformation* is to deform an object by manipulating the underlying mesh of control points. The resulting deformation controls the shape of the three-dimensional object and produces a smooth transformation. It is a powerful tool for modeling three-dimensional deformable objects introduced by [133]. A popular choice to interpolate the deformation between control points is to use trivariant *B*-spline tensor products as the deformation function. This approach was first proposed by [134]. Denote the domain of the image volumes as *Ω* = {(*x*, *y*, *z*)∣0 ≤ *x* < *X*, 0 ≤ *y* < *Y*, 0 ≤ *z* < *Z*} and let Φ denote a *n*_*x*_ × *n*_*y*_ × *n*_*z*_ uniform mesh of control points *ϕ*_*i*,*j*,*k*_. The Free Form Deformation can be written as the three-dimensional tensor product of the one-dimensional cubic *B*-splines [134].
  (6) $$\begin{array}{c} T\left(\;x,y,z\;\right)=\overset{3}{\underset{l=0}{\sum }}\;\overset{3}{\underset{m=0}{\sum }}\;\overset{3}{\underset{n=0}{\sum }}B_{l}\left(\;u\;\right)B_{m}\left(\;v\;\right)B_{n}\left(\;w\;\right)\phi_{i+l,j+m,k+n}, \end{array}$$
where *i* = ⌊*x*/*n*_*x*_⌋ − 1, *j* = ⌊*y*/*n*_*y*_⌋ − 1, *z* = ⌊*z*/*n*_*z*_⌋ − 1, *u* = *x*/*x*_*n*_ − ⌊*x*/*n*_*x*_⌋, *v* = *y*/*n*_*y*_ − ⌊*y*/*n*_*y*_⌋, *w* = *z*/*n*_*z*_ − ⌊*z*/*n*_*z*_⌋, and *B*_*l*_ represents the *l*th basis function of the *B*-spline:
  (7) B0u=1−u36,B1u=3u3−6u2+46,B2u=−3u3+3u2+3u+16,B3u=u36.
The choice of a mesh with adequate spacing is the most important question in this approach. Since the control points Φ act as parameters of the Free Form Deformation based on *B*-splines, the resolution of the mesh has a major influence on the degree of the deformation and defines the number of degrees of freedom and therefore the computational complexity.

*Nonparametric Transformation Model.* By definition,* optical flow* is image velocity approximating image motion from sequential time-ordered images. In the context of medical image processing, it has been applied to motion detection and motion compensation. The class of optical flow registration covers a very large number of methods. A detailed comparison of various optical flow methods has been given by [135].

One formulation of optical flow used as a transformation model to register pre- and intraoperative MRI images of the brain has been proposed by [49] as follows: assume the image intensity *I* of a point (*x*, *y*, *z*) on a deformable image at a time point *t* is constant for a short duration of time *δt*. If a vector (*u*, *v*, *w*) represents a velocity of the point and the intensity of the point does not change over the time *δt*, then

(8) $$\begin{array}{c} I\left(\;x,y,z,t\;\right)=I\left(\;x+u\delta t,y+v\delta t,z+w\delta t,t+\delta t\;\right). \end{array}$$

Assuming the image intensity varies smoothly with *x*,  *y*,  *z*, and *t*, (8) can be formulated with a first-order Taylor expansion.

(9) Ix,y,z,t≈Ix,y,z,t+uδt∂I∂x+vδt∂I∂y+wδt∂I∂z+δt∂I∂t.

Dividing by *δt*, the constraint equation to solve for (*u*, *v*, *w*) can be obtained:

(10) $$\begin{array}{c} I_{x}u+I_{y}v+I_{z}w+I_{t}=0, \end{array}$$

where *I*_*x*_,  *I*_*y*_,  *I*_*z*_, and *I*_*t*_ denote ∂*I*/∂*x*,  ∂*I*/∂*y*,  ∂*I*/∂*z*, and ∂*I*/∂*t*, respectively. Since the brain deforms slowly [5] after the dura is opened, brain shift can be modeled as a slow motion which can be well described with the optical flow method. However, even for the sudden deformation caused by skull and dura opening, this method is insufficient and certainly cannot be used to model deformation caused during surgical resection.

#### 5.1.2. Registration Basis

The registration basis is a measure of the alignment of the images. Usually, images can be aligned either with a feature-based approach or with an intensity-based method. The aim of feature-based registration approaches is minimizing the distance between corresponding features, such as points, lines, or surfaces in the source and target images. This means that feature-based registration approaches require the extraction of the features as well as the estimation of correspondences [136]. Since the features are extracted before the registration, segmentation errors will propagate into later stages. These errors cannot be corrected in the registration step.

While feature-based registration algorithms can reliably align boundaries, quantify the change of certain anatomical structures with high precision, and are used both for mono- and multimodality registration, intensity-based registration algorithms use the intensities throughout the whole images and therefore yield deformation values based on the image content also in regions where it is difficult to detect distinct features. The idea of intensity-based registration approaches is the optimization of a similarity metric such as Sum of Squared Differences (SSD), Normalized Cross Correlation (NCC), and normalized mutual information (NMI) measuring the degree of shared information of the image intensities. As an advantage, intensity-based methods carry out registration without any fiducial markers and without explicit segmentation of corresponding features. However, the choice of a suitable similarity measure is not trivial, especially when used for multimodality registration.

#### 5.1.3. Similarity Measure

Similarity measure is a function used to quantify the similarity between two objects. In a feature-based approach, the most intuitive similarity measure is based on feature points. Assuming that the correspondence of two given point sets **p** and **q** is known a priori, the point-based similarity measure can be defined as the squared distance of the points. However, in practice the correspondence of the points is usually unknown. Aligning surfaces can overcome this problem. Anatomically relevant surfaces are first segmented manually or automatically from the source and target images. These surfaces are then described by point clouds. Since the correspondences of the points are unknown, the Iterative Closest Point (ICP) algorithm [137] can be used, because it assumes only that a correspondence between each point in the source point set and target point set exists. The similarity measure is defined as [136]

(11) $$\begin{array}{c} S=-\underset{i}{\sum }{\left\|\;y_{i}-T\left(\;p_{i}\;\right)\;\right\|}^{2}, \end{array}$$

where

(12) yi=minqj∈q qj−Tpi2.

With correspondences estimated with (12), the point sets are registered by minimizing (11). Another similarity measure used in feature-based registration is the* chamfer similarity function.* This function was developed and described by [138] as a technique for finding the best fit of edge points from two different images by minimizing a generalized distance between them. The algorithm takes as input binary images representing the edges. A chamfer distance map is then computed corresponding to the source image. This is an image where every nonedge voxel is given a value approximating the Euclidean distance to the closest edge voxel. The target image is then superimposed onto the distance map and translated and rotated in an iterative manner until the root mean square of the voxels in the distance map corresponding to edge points in the target image is at a minimum.

The similarity measures in intensity-based approaches measure the degree of shared information of the image intensities. The simplest is the* Sum of Squared Differences* (SSD) between the intensities in the source image and the target image. The use of this similarity measure supposes that the source image and target image have the same characteristics. This is a very restrictive assumption; thus the Sum of Squared Differences is only used to register images from the same modality. A more general formulation of similarity measure is the* Normalized Cross Correlation* (NCC) which assumes a linear relationship between the two images. Because of its accuracy and robustness for aligning intermodal images as well as its invariance to changes in overlapping regions,* mutual information* has become one of the most important similarity measures. The underlying idea of this similarity measure is to interpret the feature space of the image intensities as the joint probability distribution. It makes no assumption of the relationship between image intensities in source and target images and can be seen as a metric that measures how good one image “describes” the other. To avoid any dependence on the amount of image overlap, normalized mutual information (NMI) has been suggested [136].

### 5.2. Compensation Methods Guided by Intraoperative Image Data

One strategy to compensate for intraoperative brain shift in tumor resection surgery is to register intraoperative two- or three-dimensional image data captured with iMR, iUS, LRS, or Stereo Vision with the preoperative MR data. While a standard rigid body registration is sufficient to initialize the alignment between the pre- and intraoperative MR images, a sophisticated multimodal registration must be performed as the initial step, when using iUS. This is due to the fact that the MR and US images have very different characteristics and resolution, which makes the registration more challenging. An automated rigid registration method was shown in [63] to align the three-dimensional US and MR images before performing the nonrigid registration of three-dimensional US time sequences. To more completely accommodate the nature of US images, a generalized correlation ratio is chosen as the similarity measure. Phantom study and clinical evaluation shows that the registration error is below 1.5 mm.

In order to capture the elastic deformation of the brain, a group of researchers of Vanderbilt University use the Laser Range Scanner to acquire the intensity and geometric information of the cortical surface [67, 68, 70, 77]. The process begins with the acquisition of a point cloud by standard principles of laser/camera triangulation. In addition, a digital image of the field of view is captured and the coordinates are assigned to the point cloud data [67, 68]. By using an intensity-based algorithm based on the maximization of mutual information (MI), the intraoperative LRS images are registered to the preoperative segmented cortex, which is expressed as a textured point cloud by using ray casting. Radial basis functions are used here as the local transformation model. This method, which relied on intensity-based nonrigid registration to register the two-dimensional images, is able to track the brain shift with an accuracy of 1.6 mm in a phantom study. However, since parts of the segmented cortex visible in the preoperative images may not be visible in the intraoperative images and vice-versa, the purely intensity-based registration method as proposed in [67, 68] is not very robust. Ding et al. [70] use a feature-based algorithm. Landmarks on the surface blood vessels are manually selected from the two-dimensional and three-dimensional LRS image. A Robust Point Matching (RPM) algorithm proposed in [139], which is a modified Iterative Closest Point (ICP) method using Thin Plate Splines (TPS) as the transformation model, is performed on three-dimensional LRS images. In contrast to the method proposed in [67, 68], this feature-based method is appropriate for relatively large brain displacements. The comparison of the results with 2D and 3D images based on five data sets shows that the method with 2D image data is more suitable than the one with 3D range images. The algorithm proposed in [77] differs from others in the following ways: the final deformation field is computed iteratively across spatial resolutions and different scales of transformation by creating a standard image pyramid. After the scale of the transformation is adapted for each resolution, the final deformation field is computed as the sum of deformation fields. In the phantom study and the in vivo test, this registration approach produces subpixel error, even if the differences of the images are large due to the resection. However, manual interventions to find the region of interest are still necessary for the in vivo case. Another example of a flexible surface registration approach for optical imaging, either LRS or Stereo Vision, was proposed in [69]. The approach combines image intensities, texture information, and sparse landmark matching to perform the surface registration. Although validation with one clinical data set shows that this method has a precision around 2 mm, which was in the same range as the rigid registration error of the neuronavigation system before tissue deformation [69], its robustness and the reproducibility of the result need to be proved.

To capture the deformation not only on the cortical surface, but also in the deeper brain structure, several approaches using iUS as an intraoperative modality are presented [22, 59–66]. A set of homologous landmarks in the ultrasound image volume and the MR or CT volume are used to estimate the nonlinear transformation in [59, 66]. Small circles around the ventricle cavity on a phantom [59] or fiducial balloons [66] are chosen as landmarks. TPS interpolation is performed between the landmark points. Reinertsen et al. [61, 62] presented two MR-US registration algorithms that use the segmented blood vessels from both preoperative MR angiograms (MRA) or gadolinium enhanced MR images and intraoperative Doppler US images in their work. One algorithm compares the chamfer distance map (described in [138]) of segmented blood vessels, where the vessels are segmented with a vesselness filter. The second applies the RPM algorithm based on selected points on the segmented blood vessels. A series of simulation experiments, phantom study in [61] and clinical validation of the method in [62], shows that this method is able to recover large portions of nonlinear deformations even when only a very limited region of the MR image is covered by the US acquisition. Blood vessels could be seen as powerful features for the registration of preoperative MR and intraoperative US images [62]. However, the landmarks in a feature-based approach need to be selected manually and the correspondence of the point set should be known a priori. Therefore, the registration process cannot be automated. A sophisticated method proposed in [87] makes the knowledge about the correspondence of the point set redundant. The so-called Coherent Point Drift (CPD) method aligns the feature point set as a probability density estimation, where one point set presents the Gaussian Mixture Model (GMM) centroids and the other represents the data point. The phantom study shows that this approach resolves almost 80% of brain deformation in the region of interest. An intensity-based algorithm overcomes the correspondence problem in the feature-based method. Letteboer et al. [65] show in two tumor resection cases that an intensity-based nonrigid registration method based on Free Form Deformation using *B*-splines and using normalized mutual information as the similarity measure improved the volume overlap of the tumor from an average of 76% to 96%. Another intensity-based approach has been proposed in [63, 64], where a “uniform elastic” assumption of the brain tissue is made. Instead of using nMI, the authors optimized Sum of Squared Differences (SSD) with gradient descent optimization. This algorithm is able to recover an important part of the deformations and features a smooth deformation, despite the noisy nature of the US images [63]. The validation result shows that the SSD criterion is well adapted for the registration of successive images in the time sequence [64]. In general, intensity-based US-MR nonrigid registration poses a significant challenge due to the low Signal to Noise Ratio (SNR) of the ultrasound images and different image characteristics and resolution of US and MR images. Taking these into account, Arbel et al. [60] developed a strategy to generate pseudo US images from the preoperative MR based on anatomical structures, which generate sufficiently strong acoustic signals. After anatomical structures like white matter, ventricles, and cortical grey matter are segmented from the MR image, a radial gradient operator is used to generate gradient magnitude data to simulate boundaries apparent in typical US images [60]. The nonlinear pseudo US and US image registration is performed with Normalized Cross Correlation (NCC) as a similarity measurement. A multiresolution scheme is used to speed up the registration process. Quantitative results in 12 surgical cases show correction for brain shift is up to 87% at the tumor boundary.

The intensity-based nonlinear registration method using optical flow as transformation model and multiresolution to accelerate the iterative scheme has also been adapted for monomodal MR image registration (e.g., in [49]). With this algorithm, a maximum cortical surface shift of 11 mm was estimated as well as a subsurface shift around the ventricle of 4 mm. This results are in the same range as the quantification results introduced in Section 4. The clinical validation indicated the proposed method is capable of capturing surface and subsurface shifts of the brain [49]. In order to reduce the computation time of an intensity-based nonrigid registration algorithm, Soza et al. [50, 51] introduced an intensity-based approach with computations done on graphics cards. The image data were deformed using the Free Form Deformation method and the deformation of the space is proposed with three-dimensional Bézier functions. Because this kind of Free Form Deformation contains inherent elasticity, it is a good choice for describing the movements of soft tissue [50]. Evaluation with patient data shows this approach recovers the brain deformation within a precision range of 1.5 mm–2.0 mm [50, 51]. However, in some pathological cases, Free Form Deformation is not flexible enough [51]. Also, the intensity-based registration of MR images can fail in a tumor resection case because of a lack of intensity correspondences. To avoid this problem, Hartkens et al. [52] proposed a hybrid registration algorithm, where the feature information is incorporated into the intensity-based method in order to include higher level information about the expected deformation. In this work nMI is linearly combined with the feature similarity measure. Either point or surface information is detected semiautomatically in both the reference and source images. The tissue deformation is then performed as Free Form Deformation using *B*-spline interpolation between the control points. Another approach to address the registration of pre- and postresection MR images has been proposed in [56]. In this work, the pre- and postresection MR images are first aligned rigidly to each other by maximizing the mutual information of salient structures enhanced by the joint saliency map. Difference of Gaussian (DoG) key points are then detected and clustered on the contiguous matching area of the aligned images. The displacement is then estimated by warping the clustered DoG key points with Radial Base Functions. The evaluation shows this method is able to correct the brain shift induced by small (average distance error <1 pixel) and large (average distance error <1.2 pixels) resection, while four other intensity-based registration methods failed in these cases.

### 5.3. Modeling of Intraoperative Brain Deformation

The key issue in the model-based compensation method is to model the brain and the brain shift phenomenon in an adequate way. The anatomy and physiology of the human brain show that the brain is a very complex organ consisting of grey matter, white matter, dura, pia membrane, and four ventricles which are filled with cerebrospinal fluid (CSF). The stiffness of the brain is almost the same as gel, plastic, or pasta [140–142]. To compute an accurate brain model, which can be updated intraoperatively, two essential decisions are typically made, which constitutive model should be used to describe the brain tissue and which type and size of mesh element should be used to compute the brain model.  Table 4 shows a summary of the categorization of the brain modeling issue.

#### 5.3.1. Constitutive Models

Constitutive models are used to quantify the behavior of soft tissue under loading conditions. The choice of the biomechanical model must take into account prior knowledge of patient specific brain structure and the individual dynamics of brain shift. To date, four biomechanical models have been presented in the literature: linear elastic, nonlinear elastic, viscoelastic, and biphasic. The simplest constitutive model is the linear elastic model, which assumes a linear relationship between stress and strain [53, 55, 57, 58, 82, 86, 89–97]. While linear elasticity is a very good model for bone tissue, it does not serve very well for soft tissue mechanics for two reasons. First, most soft tissues undergo strains that qualify as large deformation. Second, the relationship between stress and strain for soft tissues is generally nonlinear [143]. Therefore in theory, a nonlinear elastic model is more suitable to model brain deformation [99–101]. As a classic model for time dependent effects, the viscoelastic model [10, 102–109] is able to model the time dependency of the brain shift phenomenon because it considers the strain history in addition [16]. The model combines the aspects of both fluid behavior, which is suitable to model the CSF, and solid behavior of the brain tissue. Once we consider brain as a composition of porous solid matrices (e.g., soft tissue) with fluid (e.g., CSF) filling the pores, the biphasic model [42, 72, 76, 81, 85, 111–129] may provide an adequate constitutive model. The mechanics of this model depend both on the solid matrix deformation and on the movement of the fluid in and out of the pores during the deformation [143]. This is exactly the situation when the brain deforms under a craniotomy.

#### 5.3.2. Mesh Element

A three-dimensional biomechanical brain model is commonly computed with Finite Element (FE) analysis. It must be ensured that the complexity of the brain shift is reflected by updating the model. Thus, the quality of the mesh is an important issue in this problem. As the most elementary and flexible mesh element, tetrahedrons [10, 45, 53–55, 57, 58, 72, 76, 81, 85, 86, 90, 91, 93–97, 100–102, 105–109, 111–113, 116–129] are able to simulate the dynamical process of brain deformation and are therefore commonly used. On the other hand, as the time to estimate the brain shift in neurosurgery is limited, the Finite Element Model (FEM) of the brain should be able to be updated in real time. The speed of the FEM directly depends on the number of degrees of freedom the system has. Since the accuracy achieved with numerous tetrahedrons can be attained with only a handful of hexahedrons, hexahedral mesh elements are frequently applied [82, 92, 99–106, 108]. However, the flexibility of such a model is not as high as the FEM generated with tetrahedrons. Compromises between the flexibility and quality are pentahedral elements used in [92] as well as a combination of tetrahedrons and hexahedrons proposed in [100–102, 106, 108]. When only a two-dimensional mesh is generated, quadrilateral elements [89, 115] are sufficient.

### 5.4. Model-Based Compensation Techniques

The idea of a model-based compensation approach is to first precompute a patient specific brain model based on the preoperative MR data and then combine this model with intraoperative data to update the preoperative image.

Commonly, the widely accepted iMR technology estimates the deformation field only at sparse locations, because MR suffers from lower resolution, lack of image structure, noise, and intensity artifacts. Clatz et al. [55] and Drakopoulos et al. [58] presented a robust model driven brain shift compensation algorithm that relies on a sparse displacement field estimated with a block matching method and a linear elastic model. The same constitutive model is also used in [53, 57, 86], in which the displacement field is estimated on iMR images based on selected key features. Another example is the work from Hastreiter et al. [45, 54] where a model-based approach using adaptively refined nonlinear intensity-based registration is proposed. Since a huge number of interpolation operations must be performed in this method, the algorithms are implemented using openGL on GPUs to accelerate the image update procedure.

Since iMR is very time consuming, model driven algorithms based on iMR images often have difficulties fitting within the intraoperative time constraint. In contrast, optical imaging (LRS and Stereo Vision) is more suitable. Generally, the patient specific volumetric model is computed with preoperative MR images before the surgery. Surface images are acquired with LRS [71–76, 78, 85] or Stereo Vision [78, 81–84]. After the surface images are registered with the model nonrigidly, the volumetric model is deformed considering the boundary conditions. To obtain the deformation in deep brain structure, visible cortical surface displacement is applied directly as a boundary condition (e.g., [71, 73, 74, 81, 82]). However, the registration inaccuracy is propagated into the model updating step without any correction. To characterize the geometry and intensity properties of the brain surface in an effective way, Miga et al. [75] use a LRS system that generates three-dimensional intensity-encoded point cloud data. When compared to point-based and Iterative Closest Point registration methods, textured LRS registration results demonstrated an improved accuracy in both phantom and in vivo experiments. The volumetric deformation was calculated by analyzing a biphasic FEM. Dumpuri et al. [72, 85], Sun et al. [76], and Chen et al. [98] determined a distribution of possible conditions, such as gravity direction, CSF loss, and effect of drugs, in order to generate a deformation atlas. The optimum of the deformation predicted by the deformation atlas and measured by the cortical surface is used as the boundary condition to update the precomputed patient specific biphasic brain model. Between 70% and 80% of the shift could be corrected with this method.

## 6. Discussion

The review presented in this study shows that intraoperative MR is used widely to compensate for brain shift because it has good soft tissue resolution and neurosurgeons are familiar with MR images. In an invited review [33], Keles summarized the benefits and disadvantages of iMR. It enables image updates and the evaluation of the extent of tumor resection during surgery. Additionally, it also can be used to identify surrounding functional structures to minimize morbidity and to compensate for the effect of brain shift. Generally, iMR increases the clinical outcome of tumor resection under craniotomy. The length of hospital stay decreased from 9.4 days (when no iMRI was used) to 5.1 days using iMRI. On the other side, MR-compatible instruments are necessary in the operating room [33, 38]. The main limitation of intraoperative MR is its cost including the surgical equipment and modification of the operating room [33]. In addition, longer operation times must be accepted [25, 38]. Each intraoperative scan with iMR takes 15 min and the patient needs to be transferred between imaging position and operating position [28]. Therefore, the iMR images cannot be updated frequently. However, serial iMRI without frequent updates is not able to present and quantify the dynamic character of brain shift, because brain shift is a time dependent phenomenon affected by various forces, which magnify or neutralize each other [37]. Thus, the use of this complex, expensive, and time consuming intraoperative imaging modality [28] remains limited with respect to the compensation for brain shift.

An inexpensive way to update the intraoperative image in real time is to use iUS. Although the real time visualization of different anatomical structures of the brain such as tumor remnants, vessel structures, and skull base and the intraoperative repeatability of iUS offer considerable benefits with respect to improved intraprocedural information [30], this technology still has not gained more acceptance than intraoperative MR. This lack of adoption may be attributed to the fact that neurosurgeons are more familiar with imaging technologies such as MR and CT rather than US [30, 88]. Additionally, US images are often difficult to interpret because echogenic structures cannot reliably differentiate normal from abnormal tissue [31]. Ultrasound technology also has poor spatial and contrast resolution and suffers from artifacts or dropout from blood and air [36, 88]. The development of three-dimensional ultrasound has increased the popularity of this technology in recent years. However, one must always keep in mind that, compared to iMR, the use of iUS is not contactless, which increases the risk of an infection.

The magnitude and direction of brain shift are generally measured in two ways: either directly in the physical space of the patient or by registering the pre- and intraoperative images. The risk factors and causes of brain shift have been estimated via statistical analysis (e.g., in [11]). As shown in Section 4, intraoperative brain shift is a very complex phenomenon. Each tumor group shows unique patterns of brain shift [48]. The quantitative analysis of brain shift shows that brain shift is a small deformation, but the introduction of surgical tools and the removal of tissues near the region of interest may introduce large local deformations. The displacement field is shaped like a bowl, with the largest displacement near the center of the craniotomy [1]. The deformation on the cortical surface and deep tumor margin is larger than the midline shift, but the shift of the cortical surface is uncorrelated to the deformation of the deep tumor margin [45]. Based on the fact that the magnitude of displacement for resection cases is generally larger than that of biopsies [8], the cause of brain shift may be simplified as the following: the brain collapses under the force of gravity and fills the space previously occupied by cerebrospinal fluid (CSF) and resected tissue [8]. Although the opening of the ventricular system is related to increased brain shift [5], measuring the loss of CSF is still not sufficient to predict the magnitude of brain shift; Hartkens et al. showed that substantial deformation is not always associated with substantial CSF loss [8]. A shift of the deep tumor margin was not significantly affected by the opening of the ventricular system [5]. The direction of brain shift cannot be predicted only by prior knowledge about patient positioning and gravity, because the direction of the brain shift is not simply parallel or perpendicular to gravity, but rather a consequence of a complex interplay between the force of gravity, boundary conditions (e.g., resected regions), fluid pressure, and other forces [8]. This has consequences for the modeling of brain shift: the assumption that gravity-induced brain deformation is parallel to the gravity is not valid. Because of the high complexity and dynamic variability of the brain shift phenomenon, predictions using only a brain model in the absence of intraoperative data are difficult.

Since soft tissues deform nonlinearly, the intuitive approach to compensate for brain deformation is to register pre- and intraoperative images nonrigidly. When using iUS to acquire intraoperative image data, feature-based multimodal registration methods are commonly used because the image characteristics and resolution of preoperative MR and iUS are very different. Therefore a purely intensity-based approach fails. Another way to register MR and US images is to generate pseudo US images from MR images, resulting in an intensity-based US-US registration. Intensity-based approaches are also applied to register pre- and intraoperative MR images. However, there is a trade-off between image resolution and intraoperative time constraints. When the intraoperative MR image has the same resolution as the preoperative images, intensity-based nonrigid registration will lead to a huge number of interpolation operations. To fit the intraoperative time constraints, GPUs or cluster computers [55] are used to accelerate the registration process. When the intraoperative MR has a lower resolution, only sparse displacement fields can be estimated. A model-based approach where the intraoperative sparse data guides a patient specific Finite Element Model overcomes this problem. Combined with a FE model, optical imaging such as a Laser Range Scanner and Stereo Vision are gaining popularity. These inexpensive and contact-less modalities provide geometric and intensity information of the cortical surface, which is registered to the surface of a FE brain model in order to update the volumetric model. In our opinion, the most promising approach for volumetric brain shift compensation should make use of intraoperative information, both volume and surface, and include a model of the brain shift phenomenon. Since the blood vessels are distributed all over the cortex and in the deeper brain structure, calculating the volumetric deformation of the brain based on the deformation of the blood vessel tree could also deliver sufficient results. Another advantage of this approach is the tracking of important vessels during the surgery, providing the surgeon with additional information about blood supply of the brain. To date, only few vessel driven brain shift compensation publications (e.g., [61, 62]) have been published. The authors used intraoperative ultrasound images in their publications. The results are limited by the image quality of the ultrasound images.

The accuracy of a model-based compensation method depends on the choice of the constitutive model to describe the biomechanical behavior of the brain. Linear elastic, nonlinear elastic, viscoelastic, and biphasic models are applied, with the biphasic model used most commonly. The biphasic model describes the brain as a solid porous matrix filled with fluid which roughly matches the complex anatomical structure of the brain. In contrast, simple biomechanical models such as linear elastic models are able to predict brain deformation if they have sufficiently good boundary conditions [8]. The quality of the FE model and the computational time to update the model depend directly on the chosen mesh element. Tetrahedrons are commonly used because of their flexibility and their ability to simulate the brain shift realistically. Since the FEM meshed with tetrahedrons has a very high degree of freedom, the computation time to update this model is much higher than the model generated with hexahedrons. A compromise between flexibility and computational time is the combination of tetrahedrons and hexahedrons, where tetrahedrons are used to generate the volume undergoing large deformations such as grey and white matter and hexahedrons are used to mesh the ventricle systems which only deform slightly.

Although intraoperative brain shift is the major source of error in image-guided neurosurgery and has been a topic of interest since 1980s, comprehensive studies that quantify the complexity and time dependency are still lacking. Our findings in this review either investigate the brain deformation before and after the dura opening or measure the pre- and postresection volumetric displacement. None of these studies quantify the brain shift in the whole tumor resection surgery. Additionally, a state-of-the-art method to measure the brain shift was not found. Both direct measurement on physical space and nonrigid registration of pre- and intraoperative images are used. In the future, studies to quantify the magnitude, direction, and causes of brain shift from the beginning to the end of a neurosurgery are desirable. A further subject of studies in the future is the performance of learning based registration methods to compensate for intraoperative brain shift. It is well-known that the human brain is a highly individual organ, brain tumors differ in shape and size, and brain shift is a very complex process with various causes. Therefore, learning based registration methods can be more appropriate for correction of brain shift once enough training data are available. To date, the state-of-the-art image modalities to compensate for brain shift are iMR and iUS. The first clinical study with LRS and FEM to correct brain deformation [144] was also published recently. However, another important interventional image modality, Digital Subtraction Angiography (DSA), has not been considered, although it is less expensive than iMR and is able to provide surgeons real time intraoperative image data with anatomical structures such as blood vessels in high resolution. In our further studies, we will propose new compensation methods for brain shift based on 3D DSA images.

## 7. Conclusion

This work presents a comprehensive review of intraoperative brain shift in tumor resection surgery with the focus on clinical experience of state-of-the-art intraoperative imaging modalities and mathematical and algorithmic aspects of different compensation techniques. It can be seen as a good complement of the existing review by [13]. In total, 126 relevant papers were reviewed and 116 were categorized and discussed according to several aspects including intraoperative modality, compensation strategy, global and local transformation model, registration basis, optimization technique, similarity measure, computational platform, constitutive model, and mesh element. The categorized publications are integrated in an interactive web tool which is available on the page http://livingreview.in.tum.de/intraoperative_brain_shift/.

## Figures and Tables

**Table 1:** A summary of the clinical result by using iMRI for tumor resection.

Publication	Author	Year	Contribution	Number of patients	Result
[24]	Hadani et al.	2001	iMR development	20	Total resection of all low grade tumors
[25]	Knauth et al.	1999	Clinical usage evaluation	41	Complete resection was diagnosed in 15 cases but was performed in 31 cases with iMRI
[26]	Hall et al.	2000	iMR development	30	Complete resection in 24 cases
[27]	Hall et al.	2000	Clinical usage evaluation	30	Complete resection in 24 cases
[28]	Nimsky et al.	2001	Clinical usage evaluation	16	Complete resection in 14 cases
[29]	Black et al.	1999	Clinical usage evaluation	31	In more than one-third of the cases, tumor residual was detected with iMRI where the surgeon considered a complete removal without iMRI

**Table 2:** A summary of publications regarding measurement and quantification of intraoperative brain shift.

Publication	Author	Year	Modality	Global transformation	Registration basis	Similarity measurement	Quantification object
[1]	Maurer et al.	1998	Coagulation	Rigid	Feature-based		Magnitude, direction, risk factor
[5]	Nimsky et al.	2000	iMR	Rigid	Feature-based	Euclidean distance	Magnitude, direction, risk factor
[8]	Hartkens et al.	2003	iMR	Nonrigid	Intensity-based	Mutual information	Magnitude
[9]	Trantakis et al.	2003	iMR	Nonrigid	Intensity-based	Mutual information	Magnitude, direction
[11]	Benveniste and Germano	2005					Risk factor
[43]	Maurer et al.	1998	iMR	Nonrigid	Intensity-based	Mutual information	Magnitude
[44]	Hill et al.	1999	iMR	Nonrigid	Intensity-based	Mutual information	Magnitude, direction
[45]	Hastreiter et al.	2004	iMR	Rigid	Feature-based	Mutual information	Magnitude, direction, risk factor
[46]	Hill et al.	1997	Coagulation	Rigid			Magnitude
[47]	Letteboer et al.	2005	iUS	Rigid		Mutual information	Magnitude, direction
[48]	Dorward et al.	1998	Coagulation				Magnitude, direction

**Table 3:** A summary about the compensation techniques for brain shift.

Modality	iMR	[45, 49–58]
	iUS	[59–66]
	Laser Range Scanner	[67–78]
	Stereo Vision	[68, 69, 79–84]

Compensation strategy	Image registration based	[49–52, 56, 59–65, 67–70, 77, 79, 80]
	Model-based	[45, 53–55, 57, 58, 71–76, 78, 81–86]

Global transformation	Rigid	[56, 63, 82]
	Nonrigid	[45, 49, 52–71, 73–84, 87]

Transformation model	Thin plate spline	[59, 62, 66, 69–71, 78, 84]
	Radial based function	[56, 67, 68, 72, 77]
	Spline	[73, 74]
	Free form deformation	[50–52, 63–65]
	Optical flow	[49]

Registration basis	Intensity-based	[45, 49–51, 54–56, 60, 63–65, 67, 68, 72, 75, 77, 83]
	Feature-based	[45, 53, 54, 56–59, 61, 62, 66, 70, 71, 73, 74, 76, 78, 81, 84, 87]
	Hybrid	[52, 69, 79, 80]

Optimization technique	Gradient descent	[52, 63, 68, 72]
	Powell	[45, 50, 51, 54, 75]
	Expectation maximization	[56, 70, 71, 76, 87]
	Levenberg-Marquardt	[64, 69]
	Multiresolution	[49, 60, 65, 77]

Computational platform	GPU	[45, 50, 51]
	Cluster computer	[55]

Similarity measurement	Euclidean distance	[45, 54, 62, 70, 71, 73, 74, 76, 81]
	(Normalized) mutual information	[45, 45, 50–52, 54, 56, 65, 67, 68, 72, 75, 77, 82, 83]
	(Normalized) correlation coefficient	[53, 55, 58, 60]
	Sum of Squared Difference	[63, 64, 69]
	Chamfer similarity	[61]
	Correlation ratio	[63]
	Gaussian mixture model	[56, 87]
	Energy function	[57, 78]

Validation	Phantom	[53, 59, 61, 63–69, 72–75, 77, 79, 80, 87]
	Clinical	[45, 49–52, 54–57, 60, 62, 63, 67–72, 75, 76, 78, 81, 84]
	Animal	[63, 64]

**Table 4:** A summary of publications about brain shift modeling.

Constitutive model	Linear elastic	[53, 55, 57, 58, 78, 82, 86, 89–98]
	Nonlinear elastic	[99–101]
	Viscoelastic	[10, 102–110]
	Biphasic	[42, 72, 76, 81, 85, 111–129]

Mesh element	Tetrahedral	[10, 45, 53–55, 57, 58, 72, 76, 78, 81, 85, 86, 90, 91, 93–98, 100–102, 105–109, 111–113, 116–129]
	Quadrilateral	[89, 115]
	Pentahedral	[92]
	Hexahedral	[82, 92, 99–106, 108]

Validation	Clinical	[76, 78, 89, 91–94, 96, 97, 102, 104–106, 111–120, 125, 126, 128, 129]
	Phantom	[98, 115]
	Animal	[42, 121, 124]
