# Corrigendum to “Intraoperative Imaging Modalities and Compensation for Brain Shift in Tumor Resection Surgery”

**DOI:** 10.1155/2019/9249016

**Published:** 2019-10-01

**Authors:** Siming Bayer, Andreas Maier, Martin Ostermeier, Rebecca Fahrig

**Affiliations:** ^1^Pattern Recognition Lab, Friedrich-Alexander-University Erlangen-Nuremberg (FAU), Erlangen, Germany; ^2^Siemens Healthcare GmbH, 91301 Forchheim, Germany

In the article titled “Intraoperative Imaging Modalities and Compensation for Brain Shift in Tumor Resection Surgery” [1], there following two errors should be corrected:

1. The number of categories should be *thirteen instead of fourteen* in the following three sentences:
  - The sentence “In total, 126 papers regarding this topic are analyzed in a comprehensive summary and are categorized according to *fourteen* criteria” in the abstract section.
  - The sentence “The *fourteen* categories of interest are brain shift compensation strategy, global transformation model, local transformation model, computational platform, registration basis, optimization method, similarity metric, intraoperative modality, constitutive model type, mesh element, quantification object, validation, and treatment (the categories in [13] are physical, surgical, biological, intraoperative imaging, other, registration, and modeling. This categorization focuses on the causes of brain shift rather than on the algorithmic approaches to identify and correct for brain shift)” in the Methods and Contribution section.
  - The sentence “In total, 116 publications are grouped by the defined *fourteen* categories” in the Methods and Contribution section.
2. Reference number [132] should be changed as follows: F. L. Bookstein, “Principal warps: thin-plate splines and the decomposition of deformations,” in *IEEE Transactions on Pattern Analysis and Machine Intelligence*, vol. 11, no. 6, pp. 567–585, June 1989. doi: 10.1109/34.24792.
